# Young breast cancer patients who develop distant metastasis after surgery have better survival outcomes compared with elderly counterparts

**DOI:** 10.18632/oncotarget.15268

**Published:** 2017-02-11

**Authors:** Jingjing Wang, Jiayu Wang, Qing Li, Pin Zhang, Peng Yuan, Fei Ma, Yang Luo, Ruigang Cai, Ying Fan, Shanshan Chen, Qiao Li, Binghe Xu

**Affiliations:** ^1^ Department of Medical Oncology, Cancer Hospital, Chinese Academy of Medical Sciences and Peking Union Medical College, Beijing, China

**Keywords:** breast cancer, young age, locoregional relapse, distant metastasis, prognosis

## Abstract

To investigate the recurrence pattern and subsequent survival outcomes in young breast cancer population, 483 young patients (≤ 35) and 739 elderly patients (≥ 65), who received mastectomy or breast-conserving surgery from 2008 to 2012, were included in this study. The young population presented with a higher rate of pathologic tumor stage (*P* < 0.001), positive pathologic lymph node (*P* < 0.001), grade III tumors (*P* < 0.001), and lymphovascular invasion (*P* < 0.001). With a median follow-up of 56.5 months, young patients had a significantly lower 5-year disease-free survival (73.7% *vs* 83.4%, *P* = 0.001), while no difference in 5-year overall survival was observed (91.7% *vs* 91.7%, *P* = 0.721). The 5-year cumulative incidences of locoregional relapse (8.9% *vs* 4.3%, *P* = 0.009) and distant metastasis (18.8% *vs* 9.5%, *P* < 0.001) were significantly higher in the young population. However, for patients with distant metastasis, the survival outcomes were significantly better in the young patients (5-year overall survival since diagnosis: 60.0% *vs* 47.3%, *P* = 0.025; 5-year overall survival after recurrence: 31.0% *vs* 24.3%, *P* = 0.001). Young breast cancer patients present with more aggressive clinicopathological features and have poor prognosis compared with elderly. But young patients with distant metastasis might have better survival outcomes.

## INTRODUCTION

Breast cancer is the most common invasive cancer and the leading cause of death from cancer among women worldwide. Patients younger than 35 years of age are relatively rare, accounting for 2% - 4% of all cases diagnosed annually in the west [1–3] but much more popular in Asia [3–6]. According to the Annual Report of Cancer Statistics in Korean in 2011, 13.2% of breast cancer was < 40 years of age, and 4.7% was < 35 years of age [6].

It is believed breast cancer at a young age is associated with more aggressive biological behavior and worse prognosis than in elderly [5, 7–17], characterized by higher incidence of recurrence and higher risk of death, even when treated with more aggressive therapies [10–14]. However, instead of reporting the overall prognosis from early stage to recurrence, few studies have investigated on survival outcomes after patients develop locoregional relapse (LRR) or distant metastasis (DM). It is speculated that young patients is much more tolerable to intensive treatment therefore might have better survival after LRR or DM, despite a shorter disease-free survival (DFS) following the surgery.

In this study, we aimed to evaluate the recurrence pattern and survival outcomes following recurrence in young breast cancer patients when compared with elderly patients.

## RESULTS

### Patient characteristics

From January 2008 to December 2012, 1222 breast cancer patients were included in the study. 483 (39.5%) of total population were younger than 35 years old. As shown in Table 1, more patients had a family history of breast cancer or ovarian cancer in the young population compared with elderly (8.7% *vs* 4.1%, *P* = 0.001). The young patients presented with a higher rate of pathologic tumor stage (*P* < 0.001), positive pathologic lymph node (*P* < 0.001), grade III tumors (*P* < 0.001), and lymphovascular invasion (*P* < 0.001). The incidence of triple negative breast cancer was also higher in young patients (15.1% *vs* 12.3%, *P* < 0.001). Furthermore, young patients were more likely to receive breast-conserving surgeries (*P* < 0.001), adjuvant chemotherapy (*P* < 0.001) and radiotherapy (*P* < 0.001).

**Table 1:** Patients’ baseline characteristics

	All	Age≤35	Age≥65	χ^2^	*P*
	*N*= 1222	*N*= 483 (39.5%)	*N*= 739 (60.5%)		
Family history				11.322	0.001
Breast cancer or ovarian cancer	72	42 (8.7)	30 (4.1)		
No	1150	441 (91.3)	709 (95.9)		
Type of surgery				17.776	<0.001
Mastectomy	893	321 (66.5)	572 (77.4)		
Breast-conserving surgery	329	162 (33.5)	167 (22.6)		
Histology				4.266	0.118
In situ	76	23 (4.8)	53 (7.2)		
Invasive	1080	438 (90.7)	642 (86.9)		
Others	66	22 (4.6)	44 (6.0)		
Tumor grade				15.512	<0.001
Grade I	76	20 (5.3)	56 (7.9)		
Grade II	599	221 (58.8)	378 (65.1)		
Grade III	282	135 (35.9)	147 (25.3)		
Pathologic tumor stage				34.599	<0.001
T0	73	22 (4.6)	51 (6.9)		
T1	626	225 (46.6)	401 (54.3)		
T2	460	199 (41.2)	261 (35.3)		
T3	31	26 (5.4)	5 (0.7)		
T4	32	11 (2.3)	21 (2.8)		
Pathologic tumor stage				11.991	0.001
T0-1	699	247 (51.1)	452 (61.2)		
T2-4	523	236 (48.9)	287 (38.8)		
Pathologic node status				19.717	<0.001
N0	630	233 (48.7)	397 (61.1)		
N1	280	129 (27.0)	151 (23.2)		
N2	125	68 (14.2)	57 (8.8)		
N3	93	48 (10.0)	45 (6.9)		
Pathologic node status				16.989	<0.001
N0	630	233 (48.7)	397 (61.1)		
N1-3	498	245 (51.3)	253 (38.9)		
Primary tumor size				24.969	<0.001
≤5cm	1175	448 (92.8)	727 (98.4)		
>5cm	47	35 (7.2)	12 (1.6)		
ER				5.564	0.018
Positive	917	345 (71.4)	572 (77.4)		
Negative	305	138 (28.6)	167 (22.6)		
PgR				0.862	0.353
Positive	875	353 (73.1)	522 (70.6)		
Negative	347	130 (26.9)	217 (29.4)		
HER2 overexpression				20.770	<0.001
Yes	254	132 (27.3)	122 (16.5)		
No	968	351 (72.7)	617 (83.5)		
Molecular subtype				32.774	<0.001
ER/PgR+ and HER2-	802	277 (57.3)	525 (71.0)		
ER/PgR+ and HER2+	171	98 (20.3)	73 (9.9)		
ER/PgR- and HER2+	85	35 (7.2)	50 (6.8)		
ER/PgR- and HER2-	164	73 (15.1)	91 (12.3)		
Inflammatory breast cancer				0.133	0.715
No	1194	471 (97.5)	723 (97.8)		
Yes	28	12 (2.5)	16 (2.2)		
Lymphovascular invasion				23.199	<0.001
No	1108	414 (85.7)	694 (93.9)		
Yes	114	69 (14.3)	45 (6.1)		
Adjuvant chemotherapy				447.438	<0.001
No	541	47 (9.7)	494 (66.8)		
Anthracycline-containing chemotherapy	446	333 (68.9)	113 (15.3)		
Chemotherapy without anthracycline	235	103 (21.3)	132 (17.9)		
Adjuvant radiotherapy				238.343	<0.001
No	759	172 (35.6)	587 (79.4)		
Yes	463	311 (64.4)	152 (20.6)		
Endocrine therapy				0.062	0.803
No	321	125 (25.9)	196 (26.5)		
Yes	901	358 (74.1)	543 (73.5)		
Trautuzumab				45.465	<0.001
No	1144	424 (87.8)	720 (97.4)		
Yes	78	59 (12.2)	19 (2.6)		

Abbreviations: ER: estrogen receptor; PgR: progesterone receptor; HER2: human epidermal growth factor receptor 2.

### Survival analysis and prognostic factors

**Figure 1:**
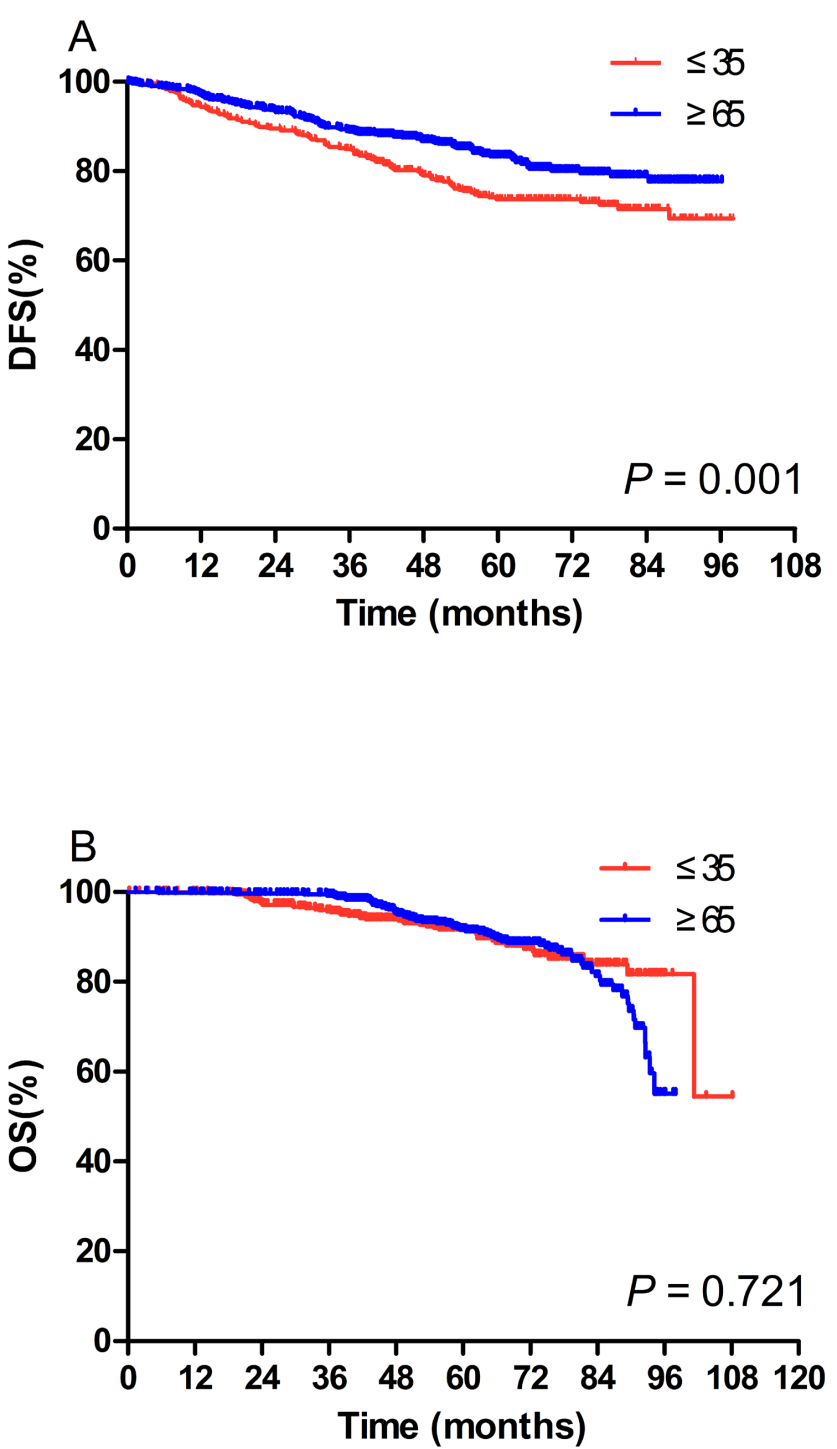
Kaplan-Meier curves of disease-free survival (DFS) (A) and overall survival (OS) (B) in the young population (N = 483) and the elderly population (N = 739) **A**. Patients in the young population had a significantly lower 5-year DFS (73.7% *vs* 83.4%, *P* = 0.001) (73.7% *vs* 83.4%, *P* = 0.001). **B**. No significant difference in 5-year OS1 was observed (91.7% *vs* 91.7%, *P* = 0.721).

After a median follow-up of 56.5 months, patients in the young population had a significantly lower 5-year DFS (Figure 1A, 73.7% *vs* 83.4%, *P* = 0.001). Nonetheless, no significant difference in 5-year OS1 was observed (Figure 1B, 91.7% *vs* 91.7%, *P* = 0.721).

In ER/PgR+ and HER2- disease, young patients were at increased risk of recurrence (5-year DFS rate: 75.2% *vs* 87.6%, *P* = 0.001) compared with elderly; whereas no difference was observed in OS1 (5-year OS1 rate: 92.5% *vs* 92.9%, *P* = 0.453). On the other hand, there was no significant difference in DFS or OS1 between two populations in ER/PgR+ and HER2+, ER/PgR- and HER2+, or ER/PgR- and HER2- disease (data not shown).

Based on multivariate survival analysis, positive axillary lymph node and large primary tumor were negatively related to DFS (*P* = 0.032, HR = 0.578, 95% CI = 0.350-0.953) (Table 2) and OS1 (*P* = 0.031, HR = 0.383, 95% CI = 0.160-0.981) (Table 3) in young patients. Similar results were observed in elderly patients (DFS: *P* < 0.001, HR = 271, 95% CI = 0.167-0.440, OS1: *P* < 0.001, HR = 0.241, 95% CI = 0.133-0.437) (Table 2 and Table 3). For young population, ER or PgR positive patients presented with longer OS1 (*P* = 0.010, HR = 2.586, 95% CI = 1.254-5.331), but have a tendency to be with shorter DFS (*P* = 0.070, HR = 0.400, 95% CI = 0.149-1.078). In the elder patients, longer OS1 was observed in patients who received adjuvant chemotherapy after surgery (*P* = 0.047, HR = 1.790, 95% CI = 1.009-3.179).

**Table 2:** Cox proportional hazards model for disease-free survival

Variables	Young group		Old group	
	HR(95%CI)	*P*	HR(95%CI)	*P*
N1-3	0.578 (0.350-0.953)	0.032	0.271 (0.167-0.440)	<0.001
Primary tumor size >5cm	0.272 (0.136-0.545)	<0.001	0.359 (0.157-0.822)	0.015
Lymphovascular invasion	0.502 (0.299-0.842)	0.009	0.788 (0.433-1.434)	0.436
ER/PgR positive	0.400 (0.149-1.078)	0.070	0.720 (0.270-1.923)	0.512
HER2 positive	0.637 (0.253-1.602)	0.338	1.299 (0.628-2.683)	0.480
Adjuvant chemotherapy	1.085 (0.374-3.144)	0.881	0.939 (0.587-1.504)	0.795
Adjuvant radiotherapy	1.621(0.986-2.666)	0.057	0.985 (0.608-1.594)	0.950
Endocrine therapy	4.021(1.378-11.729)	0.011	2.530 (1.337-4.786)	0.004
ER/PgR+ and HER2-	1.731(0.578-5.182)	0.326	0.634 (0.239-1.681)	0.360

Abbreviations: HR: hazard ratio; CI: confidential interval; ER: estrogen receptor; PgR: progesterone receptor; HER2: human epidermal growth factor receptor 2.

**Table 3:** Cox proportional hazards model for overall survival

Variables	Young group		Old group	
	HR(95%CI)	*P*	HR(95%CI)	*P*
N1-3	0.383 (0.160-0.981)	0.031	0.241 (0.133-0.437)	<0.001
Primary tumor size >5cm	0.242 (0.101-0.581)	0.001	0.241 (0.097-0.579)	0.002
Lymphovascular invasion	0.405 (0.183-0.897)	0.026	0.678 (0.327-1.405)	0.295
ER/PgR positive	2.586 (1.254-5.331)	0.010	0.931 (0.270-3.216)	0.910
HER2 positive	0.668 (0.192-2.326)	0.527	1.183 (0.490-2.856)	0.709
Adjuvant chemotherapy	2.571 (0.473-13.974)	0.274	1.790 (1.009-3.179)	0.047
Adjuvant radiotherapy	1.336 (0.576-3.098)	0.500	0.926 (0.495-1.731)	0.809
Endocrine therapy	1.819 (0.243-13.591)	0.560	1.530 (0.640-3.657)	0.339
ER/PgR+ and HER2-	2.019 (0.376-10.840)	0.412	0.493(0.153-1.585)	0.235

Abbreviations: HR: hazard ratio; CI: confidential interval; ER: estrogen receptor; PgR: progesterone receptor; HER2: human epidermal growth factor receptor 2.

### Recurrence patterns

At the last follow-up in young patients, we observed LRR in 28 (5.8%) patients, DM in 72 (14.9%), both LRR and DM in 8 (1.7%), contralateral breast cancer in 6 (1.2%), and other cancers in 1 (0.2%) patients. In the elderly, the respective recurrence rates were 2.8% (28/739), 8.1% (60/739), 0.1% (1/739), 1.5% (11/739), and 2.2% (16/739).

**Figure 2:**
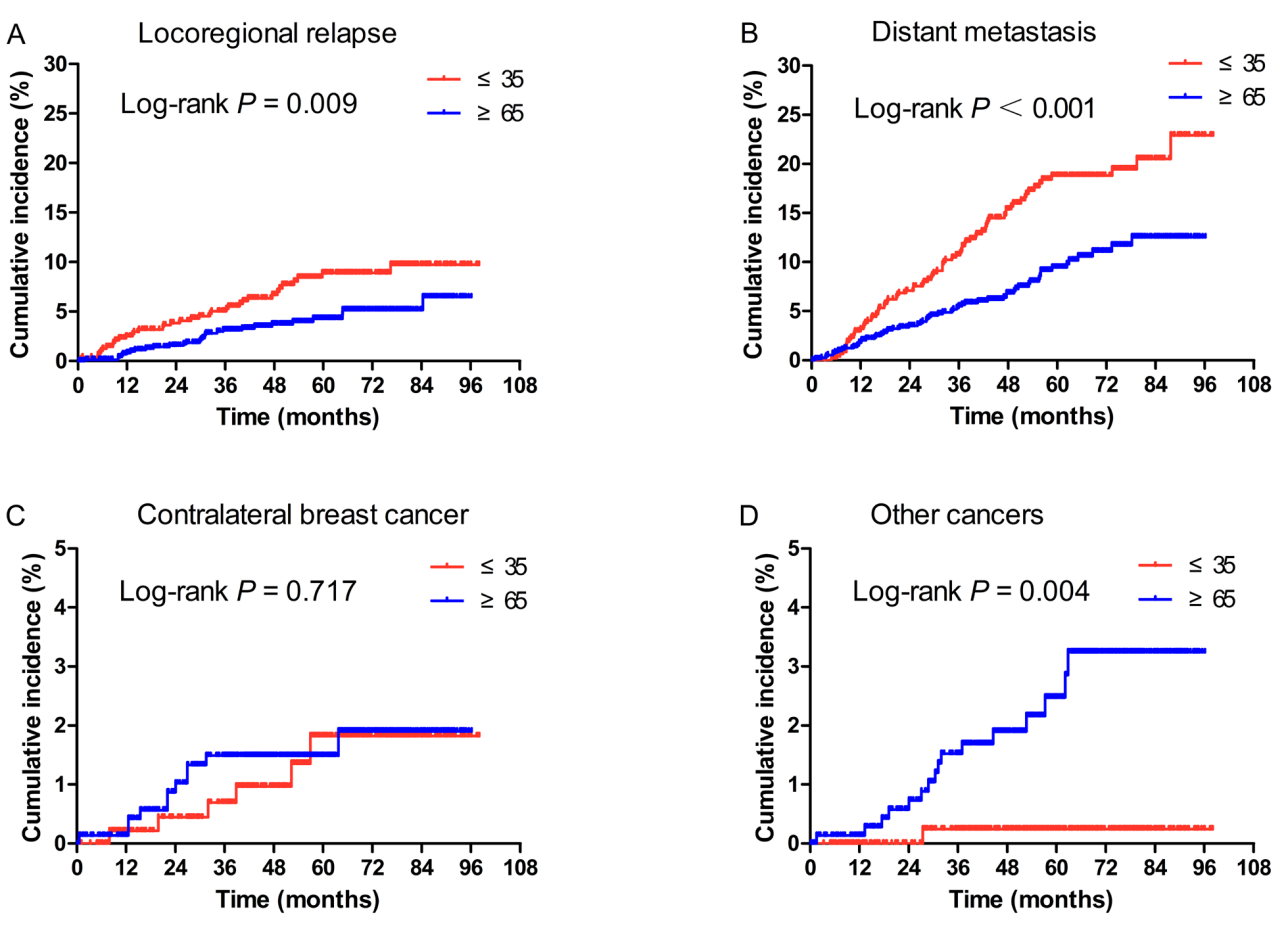
Cumulative incidence of locoregional relapse (LRR) (A), distant metastasis (DM) (B), contralateral breast cancer (C), and other cancers (D) according to age at diagnosis **A**., **B**. The 5-year cumulative incidences of LRR (Figure 2A, 8.9% *vs* 4.3%, *P* = 0.009) and DM (Figure 2B, 18.8% *vs* 9.5%, *P* < 0.001) were significantly higher in the young patients compared with elderly. **C**. The 5-year cumulative incidences of contralateral breast cancer were similar between two populations (1.8% *vs* 1.5%, *P* = 0.717). **D**. More elderly were diagnosed with other cancers in the follow-up period (2.5% *vs* 0.2%, *P* = 0.004).

As shown in Figure 2, the 5-year cumulative incidences of LRR (8.9% *vs* 4.3%, *P* = 0.009) and DM (18.8% *vs* 9.5%, *P* < 0.001) were significantly higher in the young patients compared with elderly. In contrast, more elderly were diagnosed with other cancers in the follow-up period (2.5% *vs* 0.2%, *P* = 0.004). The 5-year cumulative incidences of contralateral breast cancer were similar between two populations (1.8% *vs* 1.5%, *P* = 0.717).

### Prognosis after recurrence

**Figure 3:**
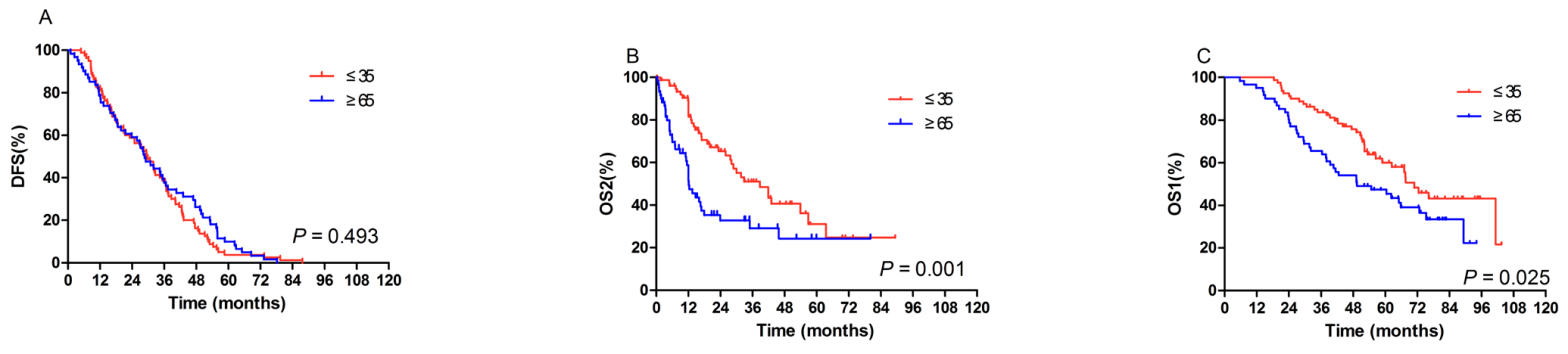
In the patients who developed distant metastasis after surgery, disease-free survival (DFS) (A), overall survival after recurrence (OS2) (B), overall survival since diagnosis (OS1) (C) according to age No difference was observed in 5-year DFS between young cohort with and elderly group. **B**., **C**. 5-year OS2 (Figure 3B, 31.0% *vs* 24.3%, 38.8 *vs* 12.1 months, *P* = 0.001) and 5-year OS1 (Figure 3C, 5-year: 60.0% *vs* 47.3%, median: 70.9 *vs* 49.4 months, *P* = 0.025) were significantly longer in the young patients compared with elderly.

In order to further explore the survival difference between young and elderly populations, we carried out stratified analysis. As shown in Figure 3, in patients with DM, 5-year OS1 (5-year: 60.0% *vs* 47.3%, median: 70.9 *vs* 49.4 months, *P* = 0.025) and 5-year OS2 (31.0% *vs* 24.3%, 38.8 *vs* 12.1 months, *P* = 0.001) were significantly longer in the young patients compared with elderly; while no difference was observed in 5-year DFS between young cohort with DM and elderly group. In addition, in patients with LRR, contralateral breast cancer, or other cancers, no difference was observed in 5-year DFS, 5-year OS2 and 5-year OS1 between age groups (data not shown).

## DISCUSSION

It has been widely believed that breast cancer at a young age is associated with a more aggressive biological behavior although there was no consensus definition for young breast cancer. Tumors in young women present with higher grade, higher T or N stage, lower differentiation, higher proliferating fraction and more vascular invasion [5, 7–17]. Azim and colleagues [18] reported that young patients had a significantly higher portion of basal-like tumors and HER2-enriched tumors. In our study, the clinicopathological characteristics of young patients were consistent with previous findings.

Following these facts, it is self-explanatory to associate young age with less favorable prognosis [5, 7–17]. Tang et al [15] demonstrated that after a follow-up of 54 months, patients < 40 years of age had inferior 5-year DFS (72% *vs* 83%, *P* < 0.01) and 5-year OS (87% *vs* 93%, *P* < 0.01) compared with those in 40-50. Consistently, lower 5-year DFS in young patients was also observed in our study (62.2% *vs* 77.8%, *P* = 0.037). In addition, a few recent studies suggested that the prognostic value of age differs by biologic subtypes. Sheridan et al [19] reported that age < 40 was associated with inferior survival within the luminal subtypes. Tang et al [15] indicated that young patients with tumors classified as luminal B type were at increased risk of poor DFS and OS; in contrast, no significant DFS or OS difference between young and elderly was observed in HER2-positive or triple negative breast cancer. Our study, on the other hand, suggested young patients with luminal A subtype had worse survival outcomes. This slight inconsistency could be attributed to the variable definitions of molecular subtypes among different studies.

Importantly, few studies have evaluated the recurrence patterns after surgery and relevant clinical implications in young breast cancer patients. Cancello et al [12] reported that patients < 35 years of age were at a higher risk to develop LRR (*P* = 0.0001) and DM (*P* = 0.0001) when compared with elderly (aged 35-50). Similar results were reported by De la Rochefordiere [20] and our group. A few studies have demonstrated that young age was an independent risk factor for increased LRR after breast-conserving surgeries in both intraductal and invasive diseases, despite given more aggressive adjuvant therapies [21–23]. Considering that higher portion of patients received breast-conserving surgery in young population in our study, the high risk of LRR in young patients could be partly attributed to the high rate of breast-conserving surgeries in our study. In addition, DM is the main recurrence pattern in young patients, much higher than LRR, justifying more intensive chemotherapy following surgery.

Despite the progress in recent years, more than 30% of patients diagnosed with early stage breast cancer will eventually progress to or relapse with advanced breast cancer [24–25]. And the overall survival for advanced breast cancer patients remains poor with a median survival ranging from 2 to 3 years [26–28]. In our study, we also compared the survival outcomes after recurrence in two populations. Better survival outcomes were observed in young patients with post-surgical DM but not with LRR. It is reasonable to speculate that young patients were able to receive more intensive treatments for better performance status and tolerability. Secondly, many patients in the elderly population died of causes other than breast cancer. Bastiaannet et al [29] investigated the relative survival (calculated as the ratio of the survival observed and the survival expected based on the corresponding general population) of elderly patients over young patients in 127,805 unselected population in Netherlands. It was reported that OS and relative survival decreased with age indicating the excess mortality in the elderly due to causes other than breast cancer. These data all suggested that in order to prolong survival, young breast cancer patients with DM should be given with more intensive treatments even the disease was incurable.

Admittedly, there were several limitations. Owing to the retrospective nature and nonrandomized design of the study, selection bias was inevitable. And the treatments were imbalanced between two populations. In HER2-positive disease, more received trastuzumab in young patients (59/132 *vs* 19/122, 44.7% *vs* 15.6%).

In conclusion, young breast cancer patients present with more aggressive clinicopathological features and have poor prognosis compared with elderly. Although they were at a higher risk to develop LRR and DM after surgery, patients with DM might have better survival outcomes.

## MATERIALS AND METHODS

### Patients

From January 2008 to December 2012, patients with operable breast cancer who received surgery at the Cancer Hospital, Chinese Academy of Medical Sciences and Peking Union Medical College were systemically reviewed. The inclusion criteria for the study were: (1) ≤ 35 years old or ≥ 65 years old; (2) newly diagnosed breast cancer; (3) available pathology report of immunohistochemistry (IHC) for estrogen receptor (ER), progesterone receptor (PgR), and human epidermal growth factor receptor 2 (HER2) status using tumor samples from core needle biopsy or surgery. The exclusion criteria were: (1) stage IV disease, bilateral breast cancer, male breast cancer, or patients complicated with other malignancies; (2) patients with incomplete medical record; (3) patients lost to follow-up immediately after treatment.

This was a retrospective observational study with information collected from hospital database. Patients’ treatments or care was not interfered throughout the course. Therefore, ethical approval and patient consents were not required.

### Treatment

Clinical evaluations at the time of this study entry included medical history and physical examination, complete blood cell count, serum biochemistry (including hepatic function, renal function, and electrolytes), electrocardiogram, bilateral breast magnetic resonance imaging or ultrasound, chest X-ray, abdominal ultrasound or computed tomography scans.

All of the mastectomies and breast-conserving surgeries were R0 resection (margin-clear resection). Adjuvant chemotherapy and radiotherapy were used at the discretion of physicians in adherence to the treatment guidelines back then, followed by endocrine therapy in cases of ER or PgR positive. Trastuzumab was recommended to HER2-positive patients but not compulsory. The status of ER, PgR and HER2 were determined by IHC. ER or PgR positive was defined as at least 1% of tumor cells with positive nuclear staining. HER2 positive was defined as 3+ by IHC or positive by fluorescent *in situ* hybridization. The study population was divided as: (1) ER/PgR+ and HER2-; (2) ER/PgR+ and HER2+; (3) ER/PgR- and HER2+; (4) ER/PgR- and HER2-.

### Statistical analysis

All data were analyzed using SPSS medical statistical software (version 15.0). DFS was defined as the duration from the diagnosis of primary breast cancer to the date of LRR or DM or last follow-up; overall survival 1 (OS1) was defined as the period from the diagnosis of primary breast cancer to the date of patient death for any cause or last follow-up; overall survival 2 (OS2) was defined as the duration from the date of LRR or DM to the date of patient death for any cause or last follow-up. Both OS and DFS were analyzed using the Kaplan-Meier method. Comparisons of OS or DFS between groups were performed using log-rank test. A two-tailed *P* < 0.05 was considered statistically significant. The Chi-squared test was performed to compare the distribution of patient characteristics between young and old patients. Multivariate analysis was done using Cox's proportional hazard regression model, and hazard ratios (HR) were presented with 95% confidential intervals (CI).
